# The Mitochondrial Hsp90 Homolog *PmTRAP1* Mediates Thermal Tolerance in the Papaya Mealybug, *Paracoccus marginatus*

**DOI:** 10.3390/insects16101064

**Published:** 2025-10-17

**Authors:** Yanting Chen, Xiaomin Zhao, Chenyu Lv, Jianwei Zhao, Mengzhu Shi, Jianwei Fu, Jianyu Li

**Affiliations:** 1 Fujian Key Laboratory for Monitoring and Integrated Management of Crop Pests, Institute of Plant Protection, Fujian Academy of Agricultural Sciences, Fuzhou 350013, China; fafucyt@126.com (Y.C.);; 2 Institute of Quality Standards and Testing Technology for Agro-Products, Fujian Academy of Agricultural Sciences, Fuzhou 350002, China

**Keywords:** papaya mealybug, Hsp90, Hsp75, mitochondrial chaperone TRAP1, heat stress

## Abstract

The papaya mealybug, *Paracoccus marginatus*, a significant invasive pest in tropical and subtropical regions, exhibits a notable capacity to withstand high-temperature stress. To elucidate the molecular basis of this thermotolerance, we investigated the role of heat shock protein 90 (Hsp90) genes in this species. The full-length cDNA sequences of three *Hsp90* genes—*PmHsp90-1*, *PmHsp90-2*, and *PmTRAP1*—were cloned, subjected to bioinformatic analysis, and their expression profiles under heat stress were detected. RNAi-mediated suppression of *PmTRAP1* was conducted to evaluate survival under extreme high-temperature conditions. The open reading frames (ORFs) of *PmHsp90-1*, *PmHsp90-2*, and *PmTRAP1* are 2175 bp, 2178 bp, and 2085 bp in length, encoding proteins comprising 724, 725, and 694 amino acids, respectively. Phylogenetic and structural analyses confirmed that PmHsp90-1 and PmHsp90-2 are cytosolic isoforms, each containing a characteristic C-terminal MEEVD motif, while PmTRAP1 was identified as the mitochondrial isoform. All three genes were significantly upregulated under heat stress. RNAi-mediated knockdown of *PmTRAP1* markedly reduced the survival rate of *P. marginatus* under extreme high temperature. These findings demonstrate that *PmTRAP1* is essential for heat tolerance in the papaya mealybug. This study provides crucial insights into the molecular mechanisms of thermal adaptation in insects and identifies *PmTRAP1* as a potential target for future research on managing insect responses to environmental stress.

## Simple Summary

The papaya mealybug, *Paracoccus marginatus*, is a major agricultural pest known for its high-temperature resilience. This study explores the molecular basis of thermal adaptation in this species by focusing on the role of heat shock protein 90 (Hsp90) genes. We cloned and characterized three *Hsp90* genes—*PmHsp90-1*, *PmHsp90-2*, and *PmTRAP1*—and analyzed their expression under heat stress. Functional analysis via RNA interference demonstrated that suppression of *PmTRAP1* significantly reduced survival under extreme high-temperature conditions. Our findings reveal the essential function of a mitochondrial chaperone in insect thermal adaptation and provide new insights into the molecular basis of environmental stress response in insects.

## 1. Introduction

The papaya mealybug, *Paracoccus marginatus* Williams and Granara de Willink (Hemiptera: Pseudococcidae), is a highly polyphagous invasive pest species. It infests over 200 host plants including economically important crops such as citrus, papaya, cassava and pepper, as well as numerous ornamentals [1]. Both nymphs and female adults damage plants through pierce-sucking feeding on stems, leaves, and fruits, causing chlorosis, defoliation, twig dieback, and reductions in fruit quality [2]. The papaya mealybug is native to Central America and has subsequently invaded numerous countries across Africa, North America, Oceania, Central and South America, and Asia [3,4]. Its spread is primarily attributed to the movement of infested plant materials and fruits, facilitating its establishment in new territories [5]. As a pest predominantly distributed in tropical and subtropical regions [6], the survival and distribution of *P. marginatus* are intrinsically linked to temperature. Therefore, understanding its molecular mechanisms for coping with high-temperature stress is fundamental to deciphering its environmental adaptability and invasive potential.

Current research on the thermal adaptability of *P. marginatus* has primarily focused on its biological traits—such as development and reproduction—and physiological responses. Studies indicate that the upper temperature threshold for egg hatching is 37 °C, while the optimal and maximum developmental temperatures for adult females are 28.4 °C and 32.1 °C, respectively [7]. Life table analyses further reveal that this pest can establish populations within the temperature range of 20–30 °C, with the highest net reproductive rate observed at 30 °C [8]. Under extreme high-temperature stress (36 °C), the activities of key antioxidant enzymes in *P. marginatus*, including peroxidase (POD), polyphenol oxidase (PPO), catalase (CAT), and superoxide dismutase (SOD), are significantly elevated, indicating an essential physiological mechanism for mitigating thermal damage [9].

Previous molecular studies have identified 12 *PmHsp70* genes in *P. marginatus*, among which *PmHsp70-11* and *PmHsp70-12* were shown to play critical roles in the heat stress response [10]. Heat shock proteins (Hsps) are known to enhance insect tolerance to various biotic and abiotic stressors [11]. Based on molecular weight, Hsps are mainly classified into six families: small heat shock proteins (sHSP), Hsp40, Hsp60, Hsp70, Hsp90, and Hsp110 [12]. Hsp90 is a highly conserved molecular chaperone that maintains protein and cellular homeostasis. Unlike chaperones involved in de novo protein folding, Hsp90 stabilizes metastable client proteins in partially folded states, facilitating their conformational maturation, stability, and assembly into functional complexes [13]. It is estimated that Hsp90 interacts with approximately 10% of the eukaryotic proteome [14]. In most eukaryotes, functional specialization of Hsp90 is achieved through compartment-specific isoforms: cytosolic Hsp90, endoplasmic reticulum (ER)-localized GRP94 (94 kDa glucose-regulated protein), mitochondrial TRAP1 (tumor necrosis factor receptor-associated protein 1), and—in plants—chloroplastic Hsp90C [15].

Accumulating evidence suggests that Hsp90 participates in various insect physiological processes, including development, reproduction, and responses to environmental stressors such as chemical exposure and thermal fluctuation [16,17,18,19]. For example, in *Anaphothrips obscurus* (Müller), *AoHsp90* expression was significantly up-regulated following heat shock treatments [20]. Similarly, in *Sitodiplosis mosellana*, *Hsp90* has been implicated in diapause maintenance and thermal stress response [21]. While studies on insect Hsp90 have provided insights into thermal responses, most have centered on cytosolic isoforms. The functions of compartment-specific isoforms, particularly the mitochondrial TRAP1, in insect thermotolerance remain poorly understood. In *Nilaparvata lugens*, *NlGRP94* is essential for oogenesis, fecundity, and late embryogenesis, whereas RNAi knockdown of *NlTRAP1* did not result in obvious phenotypic defects, nor was its expression induced by temperature stress [22]. In *Bombyx mori*, *TRAP1* promotes the proliferation of the *B. mori* nuclear polyhedrosis virus (*BmNPV*) [23]. Structural studies suggest that the N-terminal strap of *TRAP1* regulates a thermosensitive kinetic barrier during conformational cycling, implicating its role in thermal adaptation in higher eukaryotes [24]. Accordingly, the expression of *AgHsp75* (a *TRAP1* homolog) in *Aphis glycines* was induced by high temperature (36 °C) [25]. Nevertheless, the functional role of *TRAP1* in insect thermotolerance remains to be experimentally validated.

In this study, we cloned the full-length cDNA sequences of the *PmHsp90* genes from *P. marginatus* and performed comprehensive bioinformatic analyses. The expression profiles of *PmHsp90s* under different thermal stress conditions were characterized to preliminarily assess its responsiveness to temperature variations. Furthermore, RNA interference (RNAi) was employed to silence *PmTRAP1* to investigate its biological function in mediating thermal tolerance in the papaya mealybug. These results will enhance our understanding of the molecular mechanisms underlying thermal adaptation in *P. marginatus*.

## 2. Materials and Methods

### 2.1. Insect Rearing

The laboratory colony of papaya mealybug was established from individuals collected on papaya (*Carica papaya* L.) leaves on the campus of Fujian Agriculture and Forestry University (FAFU), Cangshan District, Fuzhou City, Fujian Province, China, in 2018. Since then, the population has been continuously reared on potato (*Solanum tuberosum* L.) sprouts under controlled conditions in an artificial climate chamber. The rearing conditions were maintained at 26 °C, 75% relative humidity, and a photoperiod of 14 h light/10 h darkness. For all experiments, insects were selected from this long-term colony, which had been maintained for multiple generations under the standard rearing conditions, ensuring their full acclimation to the host and environment.

### 2.2. Total RNA Extraction and cDNA Synthesis

Approximately 10 adult papaya mealybugs were used for total RNA extraction using the SteadyPure Universal RNA Extraction Kit (AG21024, Accurate Biology, Changsha, China), following the manufacturer’s protocol. The concentration and purity (OD_260_/OD_280_ ratio) of the extracted total RNA were measured using a NanoDrop 2000 spectrophotometer (Thermo Fisher Scientific, Waltham, MA, USA), and its integrity was verified by electrophoresis on a 2% agarose gel.

cDNA templates for full-length gene cloning were synthesized using a reverse transcription kit (R312-02, Vazyme, Nanjing, China) according to the following steps: First, 1000 ng of total RNA was mixed with nuclease-free water to a final volume of 8 μL, incubated at 65 °C for 5 min, and immediately placed on ice for 2 min. Then, 2 μL of 5× gDNA wiper mix was added, and the mixture was incubated at 42 °C for 2 min. Finally, 2 μL of 10× RT mix, 2 μL of Enzyme mix, 1 μL of Oligo dT primer, and 5 μL of nuclease-free water were added. After thorough vortexing and brief centrifugation, the reaction mixture was subjected to thermal cycling under the following conditions: 25 °C for 5 min, 37 °C for 45 min, and 85 °C for 5 s. The synthesized cDNA was stored at −20 °C.

### 2.3. Identification and Molecular Cloning of PmHsp90 cDNA

#### 2.3.1. Candidate Gene Screening

Members of the Hsp90 gene family were identified through integrated analysis of a previously established papaya mealybug transcriptome dataset. Initial functional annotation of transcripts was performed using multiple databases, including NCBI NR (Non-Redundant Protein), GO (Gene Ontology), COG (Clusters of Orthologous Groups), KEGG (Kyoto Encyclopedia of Genes and Genomes), Pfam, and Swiss-Prot. Candidate *PmHsp90s* were initially selected based on HSP domain homology. Redundant sequences derived from sequencing artifacts or assembly errors were eliminated via multiple sequence alignment. Subsequent validation included conserved domain identification using NCBI CDD (https://www.ncbi.nlm.nih.gov/cdd, accessed on 9 April 2025); functional module detection with SMART (https://smart.embl.de/smart/change_mode.cgi, accessed on 9 April 2025) and signature motif screening using ScanProsite (https://prosite.expasy.org/scanprosite/, accessed on 9 April 2025). This bioinformatic pipeline confirmed a unique *PmHsp90* sequence containing complete *Hsp90* characteristic domains.

#### 2.3.2. Molecular Cloning of *PmHsp90* Isoforms

Full-length cDNA sequences of three *PmHsp90* isoforms (*PmHsp90-1*, *PmHsp90-2*, and *PmTRAP1*) were amplified from cDNA templates using gene-specific primers (Table 1) designed with Oligo software (version 7.0, Molecular Biology Insights, Inc., Cascade, CO, USA). PCR reactions were conducted in 25 μL volumes containing 12.5 μL 2× Reaction Buffer, 0.5 μL dNTP Mix (10 mM each), 1.0 μL each of forward and reverse primers (10 μM), 0.5 μL DNA polymerase, 7.5 μL nuclease-free water, and 2.0 μL cDNA. Thermocycling comprised initial denaturation (95 °C, 5 min), 35 cycles of denaturation (95 °C, 15 s), isoform-specific annealing (56 °C for *PmHsp90-1*/*PmHsp90-2*; 49 °C for *PmTRAP1*; 20 s each), and extension (72 °C, 130 s), followed by final extension (72 °C, 5 min).

The target PCR product was purified using the Magen Gel Extraction Kit (D2111-03, Magen, Shanghai, China) following the manufacturer’s protocol. Purified DNA fragments were ligated into the Hieff Clone^®^ Zero TOPO-BLunt Simple Cloning Kit (10910ES20, YESEN, Shanghai, China) in a 10 μL reaction containing 2 μL insert DNA, 1 μL 10× Enhancer Solution, 1 μL vector, and nuclease-free water. After 4 min incubation on ice, the ligation mixture was transformed into DH5α competent cells (11802ES80, YESEN, Shanghai, China) via heat shock (42 °C, 45 s) following 25 min ice incubation. Transformed cells were recovered in 500 μL antibiotic-free LB medium (37 °C, 200 rpm, 60 min), concentrated by centrifugation (5000× *g*, 1 min), and plated on LB agar containing carbenicillin (100 μg/mL). Positive clones were screened after 12–16 h incubation at 37 °C. Single colonies were cultured overnight in LB-carbenicillin broth and submitted to Tsingke Biotechnology (Beijing, China) for Sanger sequencing.

#### 2.3.3. Bioinformatic Characterization of PmHsp90 Isoforms

Open reading frames (ORFs) of *PmHsp90-1*, *PmHsp90-2*, and *PmTRAP1* were predicted using the NCBI ORF Finder (https://www.ncbi.nlm.nih.gov/orffinder/, accessed on 2 April 2025). Protein properties were analyzed through soelectric point (pI) and molecular weight (Mw) prediction using ExPASy (https://web.expasy.org/compute_pi/, accessed on 3 April 2025), and subcellular localization forecasting with TargetP-2.0 (https://services.healthtech.dtu.dk/services/TargetP-2.0/, accessed on 3 April 2025).

Amino acid sequences of *PmHsp90* genes were aligned with known insect Hsp90 orthologs from *Phenacoccus solenopsis*, *Bombyx mori*, *Drosophila virilis*, *Tribolium castaneum*, *Aphis glycines*, *Spodoptera frugiperda*, and *Trichogramma chilonis* using DNAMAN (version 9.0, Lynnon Corp, Pointe-Claire, QC, Canada).

Phylogenetic analysis was performed using Hsp90 homologs from 14 insect species: *Nilaparvata lugens*, *S. frugiperda*, *T. chilonis*, *Exorista civilis*, *Zeugodacus cucurbitae*, *Cotesia vestalis*, *Pteromalus puparum*, *Loxostege sticticalis*, *Plutella xylostella*, *Mythimna separata*, *Pieris rapae*, *Bombyx mori*, *Apis mellifera*, and *P. solenopsis*. All reference sequences were retrieved from NCBI GenBank (accession numbers provided in Section 3.1). Phylogenetic reconstruction was conducted in MEGA (version 12.0, Mega Limited, Auckland, New Zealand) using the neighbor-joining method with 1000 bootstrap replicates.

### 2.4. Thermal Stress Response of PmHsp90 Expression

The expression patterns of *PmHsp90s* under high-temperature stress were investigated. Female adults were transferred into Petri dishes (90 mm in diameter) with multiple ventilation holes to ensure adequate air circulation. The dishes containing papaya mealybugs were then exposed to a range of temperature treatments in an artificial climate chamber. Based on preliminary survival assays, the mealybugs were exposed to 38 °C, 42 °C, and 46 °C for 30 min, with a control group maintained at 26 °C. All treatment temperatures were set below the previously determined lethal threshold of 48 °C (unpublished data). Three biological replicates (10 adults per replicate) were maintained per treatment. Samples were immediately flash-frozen in liquid nitrogen post-treatment and stored at −80 °C until RNA extraction.

Total RNA was extracted from samples and reverse-transcribed using the HiScript III 1st Strand cDNA Synthesis Kit (R312, Vazyme Biotech, Nanjing, China). Gene-specific qPCR primers for *PmHsp90* isoforms were designed with Oligo 7.0 (Table 1). *Pmβ-actin* was used as the endogenous control for normalization [26]. Its stability under heat stress conditions in *P. marginatus* has been empirically confirmed by its consistent Cq values in our previous gene expression analysis [10] and was verified again across all samples in the present study. Quantitative real-time PCR was performed in 20 μL reactions containing 10 μL ChamQ Universal SYBR qPCR Master Mix (Q711, Vazyme Biotech, Nanjing, China), 0.4 μL ROX2 Reference Dye, 0.4 μL each of forward/reverse primers, 2 μL cDNA template, and nuclease-free water. Amplification was conducted on a QuantStudio^TM^ 6 Pro Flex real-time PCR system (Thermo Fisher Scientific, Waltham, MA, USA) under the following protocol: 95 °C for 30 s; 40 cycles of 95 °C for 10 s and 60 °C for 30 s (annealing/extension); followed by melt curve analysis (95 °C for 15 s, 60 °C for 60 s, 95 °C for 15 s). All reactions included three technical replicates.

Relative gene expression was calculated via the 2^−ΔΔCt^ method [27]. Statistical significance (*p* < 0.05) was determined by independent samples *t*-test using SPSS (Version 27.0, IBM Corp., Armonk, NY, USA), with data visualization in GraphPad Prism (Version 8.3.0, GraphPad Software, San Diego, CA, USA).

### 2.5. Functional Validation of PmTRAP1 via RNA Interference

#### 2.5.1. Double-Stranded RNA Synthesis

T7 promoter-containing dsRNA primers targeting *PmTRAP1* (Table 1) were used to amplify template DNA followed by gel purification. Double-stranded RNA (dsRNA) was synthesized using the T7 RNA Transcription Kit (SJ004, BELONGBIO, Chengdu, China). Each 50 μL reaction contained the following components: 5 μL of 10× Transcription Buffer, 8 μL of NTP Mix, 1 μL of linear template DNA, 1 μL of RNase Inhibitor, 1 μL of T7 RNA Polymerase, and nuclease-free water up to the final volume. The reaction was incubated at 37 °C for 2 to 8 h. Subsequently, the synthesized dsRNA was purified and resuspended in DEPC-treated water at a concentration of 10,000 ng/μL.

#### 2.5.2. Microinjection and Sampling

Day-7 female adults were microinjected abdominally with 500 ng dsPmTRAP1 (experimental group) or dsGFP (control) using a Nanoject III microinjector (Drummond Scientific, Broomall, PA, USA; n = 25 adults/group). Following injection, the insects were allowed to recover for 30 min in Petri dishes containing moistened filter paper before being transferred onto fresh potato sprouts. The inoculated sprouts were then maintained under controlled conditions at 26 °C, 75% relative humidity, and a photoperiod of 14 h light/10 h darkness. Samples were collected at 24 h and 72 h post-injection, with five biological replicates per time point, each consisting of five adults. All samples were immediately flash-frozen in liquid nitrogen for subsequent analysis.

#### 2.5.3. Silencing Efficiency Validation

Total RNA extraction and cDNA synthesis were performed as previously described. *PmTRAP1* expression levels were quantified via qRT-PCR following established protocols. Silencing efficiency was calculated as

Efficiency (%) = [1 − RE_dsPmTRAP1_/RE_dsGFP_] × 100

where RE represents the relative expression of *PmTRAP1* normalized to the reference gene (*Pmβ-actin*), determined by the 2^−ΔΔCt^ method.

### 2.6. Thermal Tolerance Assay Following PmTRAP1 Silencing

The thermal tolerance of adult female papaya mealybugs was assessed following RNAi. Insects were divided into three groups: a non-injected (untreated) control, a control group microinjected with dsGFP, and an experimental group microinjected with dsPmTRAP1. At 24 h post-injection, each group was subjected to thermal stress at either 44 °C or 45 °C for 2 h in the artificial climate chamber. The selection of these temperatures was based on previous findings, in which the upper lethal temperature (ULT) for this species was determined to be 48 °C after 2 h of exposure. Therefore, 44 °C and 45 °C represent severe sublethal temperatures capable of inducing a measurable heat stress response while avoiding acute mortality in control treatments. Following heat exposure, mealybugs were transferred to fresh potato sprouts and allowed to recover for 24 h under standard rearing conditions. Mortality was assessed by gentle probing of appendages and cephalic regions with a soft brush under a stereomicroscope; individuals showing no voluntary movement were recorded as dead. Each temperature treatment included five biological replicates (10 adults/replicate). Differences in survival rates among the three treatment groups were analyzed using one-way ANOVA followed by Fisher’s LSD for post hoc comparisons in SPSS 25.0, with a significance level of *p* < 0.05.

## 3. Results

### 3.1. Characterization of PmHsp90 Isoforms in P. marginatus

The ORFs of *PmHsp90-1*, *PmHsp90-2*, and *PmTRAP1* measured 2718 bp, 2712 bp, and 2088 bp, encoding 725, 723, and 695 amino acid residues, respectively. The nucleotide sequences of *PmHsp90-1*, *PmHsp90-2*, and *PmTRAP1* have been deposited in the GenBank database under accession numbers PX400431, PX400432, and PX400433, respectively. Predicted molecular weights were 177.1 kDa, 177.8 kDa, and 172.3 kDa with isoelectric points (pI) of 4.94, 4.91, and 4.98.

TargetP-2.0 subcellular localization predicted cytosolic localization for PmHsp90-1 and PmHsp90-2, while PmTRAP1 localized to mitochondria. This indicates *PmHsp90-1* and *PmHsp90-2* belong to cytosolic Hsp90 family, whereas *PmTRAP1* represents a mitochondrial Hsp (Hsp75/TRAP1).

**Figure 1:**
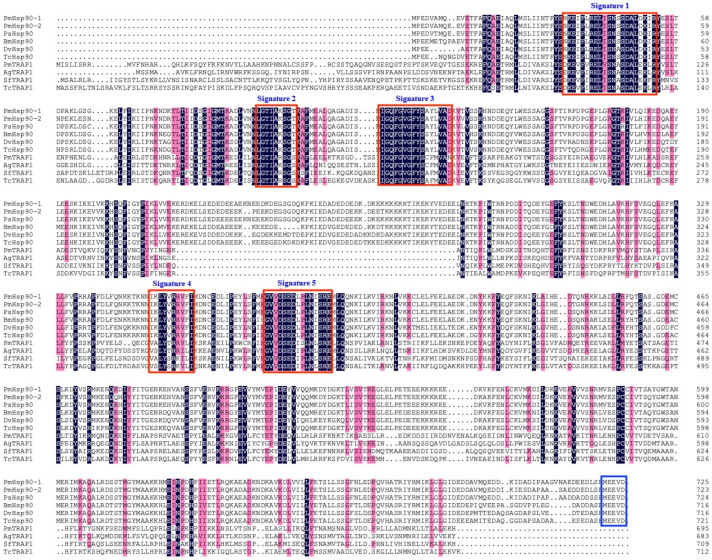
Multiple amino acid sequence alignments of Hsp90 from *Paracoccus marginatus* and other insects. PsHsp90: *Phenacoccus solenopsis*; BmHsp90: *Bombyx mori*; DvHsp90: *Drosophila virilis*; TcHsp90: *Tribolium castaneum*; AgTARP1: *Aphis glycines*; SfTARP1: *Spodoptera frugiperda*; TcTARP1: *Trichogramma chilonis*. The red box highlights conserved signatures diagnostic of the Hsp90 protein family. The blue box highlights the characteristic C-terminal ‘MEEVD’ motif. Residues shaded with a dark blue background are identical across all sequences; those with a pink background represent similar residues with a consensus level exceeding 75%.

Amino acid sequence alignment of PmHsp90s with known Hsp90s from other species revealed three highly conserved domains within all PmHsp90 family members: the N-terminal domain (NTD), middle domain (MD), and C-terminal domain (CTD) (Figure 1), despite variations in their genomic organization. Specifically, three conserved family signatures were identified within the NTD: signature 1 (NKEIFLRELISNSSDALDKIR), signature 2 (LGTIAKSGT), and signature 3 (IGQFGVGFYSAYLVAD). Two additional family signatures were located in the MD: signature 4 (IKLYVRRVFI) and signature 5 (GIVDSEDLPLNISRE). Furthermore, the C-terminal domain of both PmHsp90-1 and PmHsp90-2 contained the conserved MEEVD motif, characteristic of cytosolic Hsp90 proteins.

**Figure 2:**
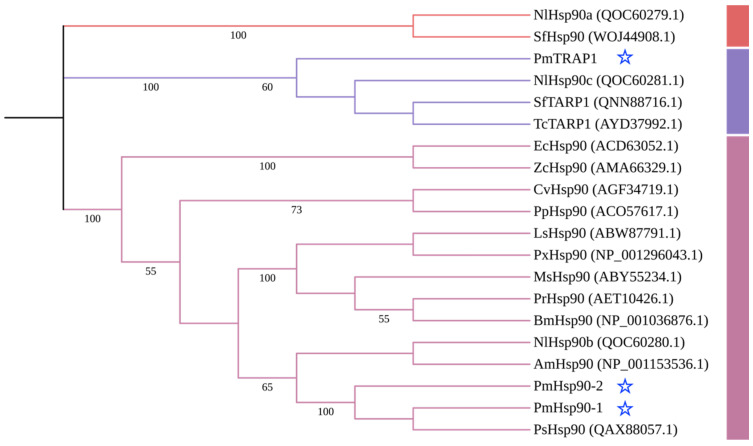
Phylogenetic analysis of Hsp90 proteins from *Paracoccus marginatus* and other insects using the Neighbor-Joining method. PmHsp90 proteins from *P. marginatus* are highlighted with blue stars. Colored bars on the right represent the major gene clades: GRP94, TRAP1, and Hsp90. NlHsp90a (QOC60279.1), NlHsp90b (QOC60280.1), NlHsp90c (QOC60281.1): *Nilaparvata lugens*; SfHsp90 (WOJ44908.1), SfTARP1 (QNN88716.1): *Spodoptera frugiperda*; TcTARP1 (AYD37992.1): *Trichogramma chilonis*; EcHsp90 (ACD63052.1): *Exorista civilis*; ZcHsp90 (AMA66329.1): *Zeugodacus cucurbitae*; CvHsp90 (AGF34719.1): *Cotesia vestalis*; PpHsp90 (ACO57617.1): *Pteromalus puparum*; LsHsp90 (ABW87791.1): *Loxostege sticticalis*; PxHsp90 (NP 001296043.1): *Plutella xylostella*; MsHsp90 (ABY55234.1): *Mythimna separata*; PrHsp90 (AET10426.1): *Pieris rapae*; BmHsp90 (NP 001036876.1): *Bombyx mori*; AmHsp90 (NP 001153536.1): *Apis mellifera*; PsHsp90 (QAX88057.1): *Phenacoccus solenopsis*.

Phylogenetic analysis revealed distinct clustering patterns for PmHsp90 isoforms. The cytoplasmic isoforms PmHsp90-1 and PmHsp90-2 formed a highly supported monophyletic clade with PsHsp90 from *P. solenopsis*. This clade then clustered with NlHsp90b from *N. lugens* and AmHsp90 from *A. mellifera*. In contrast, PmTRAP1, classified within the mitochondrial TRAP1 subfamily based on its grouping with established insect TRAP1 orthologues (SfTRAP1 from *S. frugiperda*, TcTRAP1 from *T. castaneum*, and NlHsp90c (NlTRAP1) from *N. lugens*), was phylogenetically distinct (Figure 2).

### 3.2. Thermal Induction of PmHsp90 Isoform Expression

**Figure 3:**
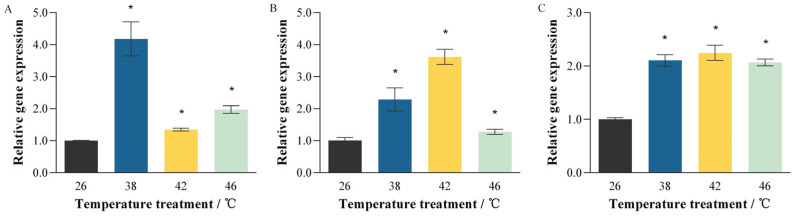
Relative expression levels of *PmHsp90* genes in *Paracoccus marginatus* under heat stress. Expression was quantified at 38 °C, 42 °C, and 46 °C relative to the 26 °C control (set as 1). (**A**) *PmHsp90-1*, (**B**) *PmHsp90-2*, (**C**) *PmTRAP1*. Asterisks indicate significant differences versus control (Student’s *t*-test: *p* ≤ 0.05). Error bars represent ± standard error (SE).

Heat treatments at 38 °C, 42 °C, and 46 °C significantly upregulated *PmHsp90* gene family expression in *P. marginatus*, though with distinct isoform-specific responses (Figure 3). Specifically, *PmHsp90-1* expression exhibited a significant 4.18-fold increase at 38 °C compared to the control (t = 7.29, df = 4, *p* = 0.002), with more modest yet significant increases of 1.35-fold at 42 °C (t = −8.090, df = 4, *p* = 0.001) and 1.97-fold at 46 °C (t = −8.090, df = 4, *p* = 0.001) (Figure 3A). *PmHsp90-2* was significantly induced at 38 °C (2.28-fold; t = −3.463, df = 4, *p* = 0.026) and 42 °C (3.61-fold; t = −9.892, df = 4, *p* = 0.001), but remained unchanged at 46 °C (Figure 3B). *PmTRAP1* demonstrated pan-thermal responsiveness with significant upregulation across all temperatures: 2.10-fold at 38 °C (t = −10.275, df = 4, *p* = 0.001), 2.24-fold at 42 °C (t = −8.526, df = 4, *p* = 0.001), and 2.06-fold at 46 °C (t = −15.732, df = 4, *p* < 0.001) (Figure 3C). Given its consistent and substantial induction at all three temperatures tested, *PmTRAP1* was selected for functional validation.

### 3.3. RNAi Silencing Efficiency of PmTRAP1 in P. marginatus

**Figure 4:**
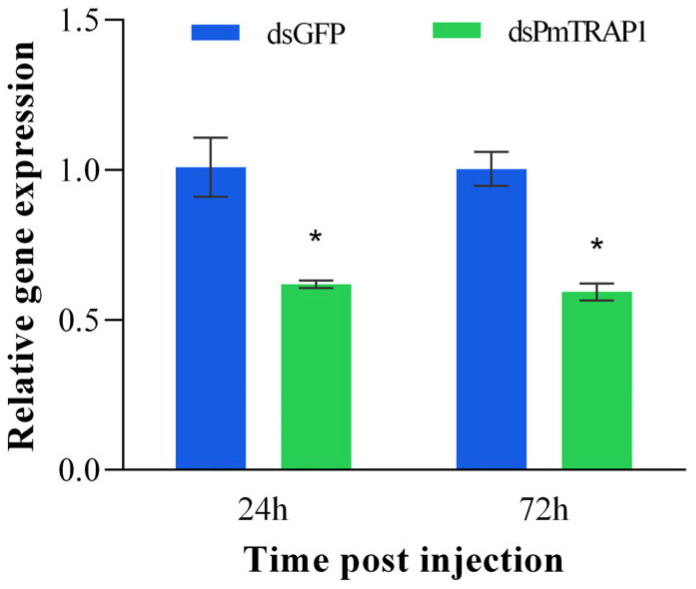
Silencing efficiency of *PmTRAP1* following dsRNA injection. Relative expression levels of *PmTRAP1* at 24 h and 72 h post-injection in dsPmTRAP1-treated groups compared to dsGFP controls. Expression in dsGFP control group was normalized to 1. Asterisks indicate significant differences versus dsGFP control group (Student’s *t*-test: *p* ≤ 0.05). Error bars represent ± standard error (SE).

Female adults injected with dsPmTRAP1 exhibited significant downregulation of target gene expression compared to dsGFP controls at both post-injection timepoints: 38.76% silencing efficiency at 24 h (t = 3.914, df = 4, *p* = 0.017) and 40.86% at 72 h (t = 6.472, df = 4, *p* = 0.003), confirming successful and persistent RNAi-mediated suppression (Figure 4).

### 3.4. Impact of PmTRAP1 Silencing on Thermal Tolerance

**Figure 5:**
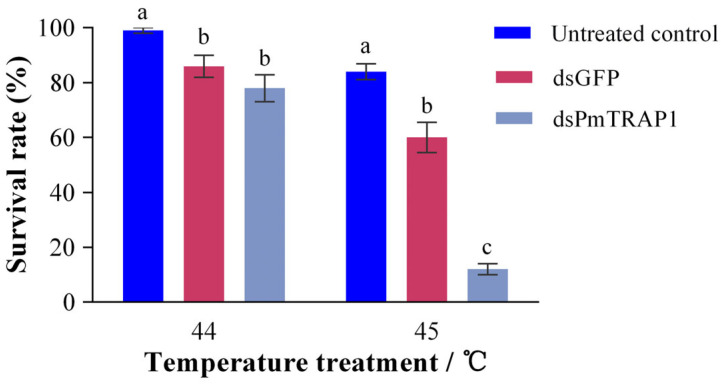
Survival response of *Paracoccus marginatus* to heat stress following RNAi-mediated suppression of *PmTRAP1*. Survival rates of adult mealybugs after 2 h exposure to 44 °C and 45 °C. Insects were either untreated (non-injected control), injected with dsGFP or injected with dsPmTRAP1. Different lowercase letters above bars indicate statistically significant differences among treatments for each temperature (one-way ANOVA followed by Fisher’s LSD, *p* < 0.05). Error bars represent ± standard error (SE; n = 5 biological replicates).

Following RNAi-mediated silencing, all microinjected insects (in both dsGFP and dsPmTRAP1 groups) survived the 24 h recovery period under standard conditions prior to heat exposure. When these insects were subsequently subjected to thermal stress, the dsGFP-injected group exhibited significantly higher mortality than the non-injected untreated control at both temperatures (44 °C: *p* = 0.029, 45 °C: *p* = 0.001; Figure 5). At 44 °C, survival rates did not differ significantly between *PmTRAP1*-silenced and dsGFP-injected control groups (*p* = 0.152; Figure 5). However, exposure to the more severe temperature of 45 °C resulted in significantly reduced survival in the *PmTRAP1*-silenced group compared to both the dsGFP-injected and untreated control groups (*p* < 0.001; Figure 5).

## 4. Discussion

*P. marginatus* is a major invasive insect species predominantly distributed in tropical and subtropical regions, demonstrating notable thermotolerance. Hsp90 plays a crucial role in mediating insect responses to various biotic and abiotic stresses [28]. The characterization of the Hsp90 gene family in *P. marginatus* reveals a system of compartment-specific chaperones involved in heat stress response. The presence of two cytosolic isoforms (*PmHsp90-1*, *PmHsp90-2*) alongside a mitochondrial homolog (*PmTRAP1*), and the marked upregulation of all three genes under thermal stress shows their involvement in *P. marginatus*’ thermal stress response. Furthermore, the role of the mitochondrial gene *PmTRAP1* in thermal adaptation was functionally validated using RNA interference technology.

Based on subcellular localization, *Hsp90* genes can be classified into three categories: cytosolic, endoplasmic reticulum (GRP94), and mitochondrial (TRAP1) [15]. In this study, only the cytosolic and mitochondrial Hsp90 genes were successfully cloned from *P. marginatus*, while the ER-localized GRP94 was not identified. This result is consistent with the number of *Hsp90* genes documented in some insects such as *N. lugens* and *B. mori* [22,23,29,30]. However, the compositional pattern differs; in both *N. lugens* and *B. mori*, one cytosolic *Hsp90*, one *GRP94*, and one *TRAP1* have been reported, whereas in *P. marginatus*, no *GRP94* homolog was detected. In contrast, certain insect species, including *A. obscurus* [20] and *S. frugiperda* [31], have been found to possess only one or two *Hsp90* genes, which may result from gene loss or limitations in transcriptome assembly completeness. Similarly, no *GRP94* homologous gene was identified in the transcriptomic data of *P. marginatus* in this study.

Multiple sequence alignment revealed that all three identified PmHsp90 proteins contain the five signature motifs conserved in the Hsp90 family, as well as the three conserved domains (NTD, MD, and CTD) consistent with the canonical Hsp90 architecture reported in other species [32,33]. Each domain serves a specialized function; the NTD is responsible for ATP binding, the MD facilitates client protein interactions, and the CTD mediates dimerization through specific structural motifs [13]. The two cytosolic isoforms, PmHsp90-1 and PmHsp90-2, possess a C-terminal MEEVD motif, which is typical of cytosolic Hsp90s and consistent with known localization patterns [13]. Phylogenetic analysis further supported their functional classification; PmHsp90-1 and PmHsp90-2 clustered with cytosolic NlHsp90 (NlHsp90b) from *N. lugens*, whereas PmTRAP1 grouped with mitochondrial NlHsp90c (NlTRAP1) from the same species. This independent evolutionary for TRAP1 could explain its significant sequence divergence (e.g., sharing only ~46% identity with cytosolic Hsp90 in *N. lugens*) [22] and distinct structural features, such as the absence of a charged linker domain between the NTD and MD and a C-terminal EEVD motif, which may account for its specialized role in the mitochondrial environment, including its temperature-sensitive ATPase activity [34]. These findings demonstrate that Hsp90 from distinct subcellular compartments exhibit high evolutionary conservation, reflecting their specialized functions in organelle-specific protein homeostasis.

As ectothermic organisms, insects are highly sensitive to ambient temperature, which profoundly influences their growth, development, lifespan, and reproduction. Current climate change, characterized by increasing global mean temperatures and a rising frequency of extreme heat events, poses substantial challenges to ectothermic insects [35,36]. Heat shock proteins play a crucial role in facilitating the appropriate folding and functional maintenance of proteins under stress conditions, thereby protecting organisms from or mitigating damage [37]. In this study, the expression of three *PmHsp90* genes in *P. marginatus* was up-regulated under thermal stress, indicating that the *PmHsp90* gene family is responsive to high-temperature stress. Notably, *PmHsp90-1* showed significant up-regulation at 46 °C, whereas *PmHsp90-2* did not. This differential expression pattern indicates that even isoforms from the same gene family with the same subcellular compartment can display distinct thermal response thresholds and dynamics The Hsp induction is not triggered by a fixed temperature set point, but rather depends on both stress intensity and duration. Such functional specialization has also been observed in other insects. For example, in *S. frugiperda*, the expression of *SfHsp90s* was significantly elevated after exposure to an extreme temperature of 45 °C, showing a transient expression pattern that increased and then decreased with prolonged treatment time [31]. Moreover, thermal stress significantly induced the expression of *Aohsp90* in *A. obscurus*, although expression declined with further temperature increases [20]. It is noteworthy that the *Hsp90* genes reported in these previous studies are all cytosolic or ER forms. In contrast, the present study demonstrates that the mitochondrial isoform *PmTRAP1* is also up-regulated under heat stress. This finding implies that, in addition to cytosolic and endoplasmic reticulum pathways, the mitochondrial pathway is involved in the heat stress response in insects, thereby expanding our understanding of the regulatory network mediated by the Hsp90 family in insect thermal adaptation.

The consistent upregulation of *PmTRAP1* under high-temperature stress is particularly notable, especially in light of its well-documented functions in other species. In humans, *TRAP1* has been extensively studied and is known to play important roles in tumor cell growth [38,39]; however, its function in insects remains relatively limited. Previous studies have demonstrated that *TRAP1* can protect cells from oxidative stress and apoptosis [40]. In *Drosophila*, knockdown of *TRAP1* resulted in mitochondrial dysfunction and increased sensitivity to heat and drug stress [41], whereas overexpression of *TRAP1* extended healthspan by enhancing stress resistance, locomotor activity, and fertility [42]. Functionally, TRAP1 differs from cytosolic Hsp90s by lacking the charged linker and C-terminal EEVD motif, and its ATPase activity is uniquely modulated by temperature and divalent cations such as Mg^2+^ and Ca^2+^ [34]. Given that mitochondria operate at temperatures approaching 50 °C under physiological conditions, which is significantly higher than that of the surrounding cytosol (37 °C) [43]. The heat-induced expression of *PmTRAP1* suggests it may act as a holdase under thermal stress, maintaining client proteins in a folding-competent state to safeguard mitochondrial proteostasis. However, studies on the response of TRAP1 to thermal stress remains scarce, with available evidence confined to only a few species. For instance, in *N. lugensm*, the expression of NlTRAP1 was not induced by temperature [22]. In contrast, in *A. glycine*, the expression of *AgHsp75* (a TRAP1 homolog) was up-regulated under both high temperature and imidacloprid exposure [25]. A similar absence of temperature responsiveness was observed in a non-insect species, the mollusk *Chlamys farreri*, where *CfTRAP1* expression showed no temperature dependence [44]. Notably, our findings demonstrated that the expression of *PmTRAP1* is clearly regulated by temperature.

The critical role of *PmTRAP1* in thermotolerance was confirmed through RNAi-mediated knockdown. Under extreme heat stress (45 °C), *PmTRAP1* knockdown individuals exhibited significantly higher mortality compared with both dsGFP-injected and non-injected controls, indicating its essential function. Notably, no significant effect was observed at the sublethal temperature of 44 °C, suggesting that *PmTRAP1* becomes indispensable only under severe thermal stress approaching the organism’s physiological limit. Our results imply that under extreme heat, *PmTRAP1* is vital for survival. While the underlying mechanism in papaya mealybug requires further investigation, this essential function may involve maintaining mitochondrial integrity, as TRAP1 deficiency has been shown to cause mitochondrial dysfunction and reduce thermotolerance in *Drosophila melanogaster* [41]. Therefore, *PmTRAP1* serves as a crucial protective component in the papaya mealybug’s thermal response under threshold conditions.

## 5. Conclusions

In conclusion, this study provides the first molecular and functional characterization of the Hsp90 gene family in *P. marginatus*. We successfully identified and cloned two cytosolic isoforms (*PmHsp90-1*, *PmHsp90-2*) and a mitochondrial isoform (*PmTRAP1*). Our results indicate that all three *PmHsp90* genes are responsive to high-temperature stress. Crucially, we have established through RNAi that the mitochondrial isoform, *PmTRAP1*, is indispensable for survival under extreme heat stress. These findings significantly expand our understanding of the molecular mechanisms underpinning thermal adaptation in this invasive pest, highlighting the importance of compartment-specific Hsp90 function. While *PmTRAP1* is a key protector against acute thermal stress, its role under chronic or fluctuating field-relevant temperatures needs further investigation. Furthermore, given the pleiotropic function of *Hsp90* family genes, future studies should explore the roles of *PmHsp90s* in adapting to other stressors, such as insecticide exposure. Overall, this work establishes a solid molecular foundation for understanding environmental adaptation in this significant invasive pest.

## Figures and Tables

**Table 1:** Primers used for gene cloning, quantitative real-time PCR (qRT-PCR), and double-stranded RNA (dsRNA) synthesis in this study.

Gene	Forward Primer (5′-3′)	Reverse Primer (5′-3′)	Usage
*PmHsp90-1*	ATGCCGGAAGACGTAGCTATG	TCAATCGACTTCTTCCATTCT	cDNA sequence cloning
*PmHsp90-2*	ATGCCTGAAGACGTAGCAATG	TTAATCGACCTCTTCCATTCT	
*PmTRAP1*	ATGATTAGTTTAATCAGTCGT	TTAATGTTTTTCCAAAGCTAA	
*PmHsp90-1*	AGACAGAAAGCCGAAGCAGAT	CTGCTTCGGCTTTCTGTCTCA	Fluorescence quantitative PCR
*PmHsp90-2*	GAAGCCAACTCCGAATTGACCA	GGTATGACGAAAGCCGACCTTG	
*PmTRAP1*	GGTATGACGAAAGCCGACCTTG	GCTGTAGAAACCGACACCGAAT	
*Pmβ-actin*	CATCCTGCGTTTGGATTTAG	TCCAAAGCAACATAGCACAAT	
*PmTRAP1*	TAATACGACTCACTATAGG AAGGAAACGTATCTGAAGAGC	TAATACGACTCACTATAGG CCAATAATACCCCTTTGCTTC	dsRNA synthesis
*GFP*	AATACGACTCACTATAGGGCACAAGTTCAGCGTGTCC	TAATACGACTCACTATAGGGGGTGCTCAGGTAGTGGTT	

## Data Availability

The original contributions presented in this study are included in the article. Further inquiries can be directed to the corresponding author.

## References

[1] Miller D.R., Miller G.L. (2002). Redescription of *Paracoccus marginatus* Williams and Granara de Willink (Hemiptera: Coccoidea: Pseudococcidae), including descriptions of the immature stages and adult male. Proc. Entomol. Soc. Wash..

[2] Myrick S., Norton G.W., Selvaraj K.N., Natarajan K., Muniappan R. (2014). Economic impact of classical biological control of papaya mealybug in India. Crop Prot..

[3] Seni A., Chongtham S. (2013). Papaya mealybug *Paracoccus marginatus* williams & granara de willink (Hemiptera: Pseudococcidae), a current threat to agriculture—A review. Agric. Rev..

[4] CABI Crop Protection Compendium. http://www.cabi.org/cpc.

[5] Tanwar R.K. (2010). Papaya mealybug and its management strategies. Technol. Bull (Natl. Cent. Integr. Pest Manag.).

[6] Chen Y., Shi M., Fu J., Zhao Z., Liu W., Li J. (2023). Prediction of potential suitable habitats for *Paracoccus marginatus* in China under climate change conditions. J. Plant Prot..

[7] Amarasekare K.G., Chong J.H., Epsky N.D., Mannion C.M. (2008). Effect of temperature on the life history of the mealybug *Paracoccus marginatus* (Hemiptera: Pseudococcidae). J. Econ. Entomol..

[8] Amutha M., Dharajothi B. (2016). Life table and population parameters of *Paracoccus marginatus* at varying temperatures on cotton. Indian J. Plant Prot..

[9] Chen Q., Liang X., Wu C.-l., Wang Y.-r., Zhao H.-p., Chen Q. (2020). Influence of different temperatures on protective enzyme activities of *Paracoccus marginatus* (Hemiptera: Pseudococcidae). Genom. Appl. Biol..

[10] Chen Y., Zhao J., Shi M., Ruan F., Fu J., Liu W., Li J. (2024). Characterization and expression patterns of heat shock protein 70 genes from *Paracoccus marginatus* in response to temperature and insecticide stress. Agriculture.

[11] Banfi D., Bianchi T., Mastore M., Brivio M.F. (2025). The role of heat shock proteins in insect stress response, immunity, and climate adaptation. Insects.

[12] Morimoto R. (1993). Cells in stress: Transcriptional activation of heat shock genes. Science.

[13] Abdullah H., Marwan E.S., Hassan N. (2018). The *Hsp90* family: Structure, regulation, function, and implications in health and disease. Int. J. Mol. Sci..

[14] Zhao R., Houry W.A. (2007). Molecular interaction network of the *Hsp90* chaperone system. Adv. Exp. Med. Biol..

[15] Johnson J.L. (2012). Evolution and function of diverse *Hsp90* homologs and cochaperone proteins. Biochim. Biophys. Acta..

[16] Ding J.H., Zheng L.X., Chu J., Sheng S. (2021). Characterization, and functional analysis of *Hsp70* and *Hsp90* gene families in *Glyphodes pyloalis* (Walker) (Lepidoptera: Pyralidae). Front. Physiol..

[17] Garcia-Gomez B., Sánchez T.A., Cano S.N., Nascimento N.A.D., Bravo A., Soberón M. (2023). Insect chaperones *Hsp70* and *Hsp90* cooperatively enhance toxicity of *Bacillus thuringiensis Cry1A* toxins and counteract insect resistance. Front. Immunol..

[18] Will T., Schmidtberg H., Škaljac M., Vilcinskas A. (2017). Heat shock protein 83 plays pleiotropic roles in embryogenesis, longevity, and fecundity of the Pea Aphid *Acyrthosiphon pisum*. Dev. Genes Evol..

[19] Xiong W., Zhai M., Yu X., Wei L., Mao J., Liu J., Xie J., Li B. (2017). Comparative RNA-sequencing analysis of ER-based HSP90 functions and signal pathways in *Tribolium castaneum*. Cell Stress Chaperones.

[20] Guo X.J., Feng J.N. (2018). Comparisons of expression levels of heat shock proteins (*Hsp70* and *Hsp90*) from *Anaphothrips obscurus* (Thysanoptera: Thripidae) in polymorphic adults exposed to different heat shock treatments. J. Insect Sci..

[21] Cheng W., Li D., Wang Y., Liu Y., Zhu-Salzman K. (2016). Cloning of heat shock protein genes (*hsp70*, *hsc70* and *hsp90*) and their expression in response to larval diapause and thermal stress in the wheat blossom midge, *Sitodiplosis mosellana*. J. Insect Physiol..

[22] Chen X., Li Z.D., Dai Y.T., Jiang M.X., Zhang C.X. (2020). Identification and characterization of three heat shock protein 90 (*Hsp90*) homologs in the Brown planthopper. Genes.

[23] Wang X., Zhang Y., Fei S., Awais M.M., Sun J. (2021). Heat Shock Protein 75 (*TRAP1*) facilitate the proliferation of the *Bombyx mori* nucleopolyhedrovirus. Int. J. Biol. Macromol..

[24] Partridge J.R., Lavery L.A., Elnatan D., Naber N., Cooke R., Agard D.A. (2014). A novel N-terminal extension in mitochondrial *TRAP1* serves as a thermal regulator of chaperone activity. eLife.

[25] Wang X., Li S., Yao L., Liu Y., Chen Y., Yang H., Fan D. (2020). The stress-induced expression of the *Aghsp75* gene in *Aphis glycines* in response to imidacloprid and temperature stress. Chin. J. Biol. Control.

[26] Liang X., Chen Q., Wu C., Liu Y., Han Z., Wu M. (2021). Reference gene selection for analyzing the transcription patterns of two fatty *acyl-CoA* reductase genes from *Paracoccus marginatus* (Hemiptera: Pseudococcidae). J. Insect Sci..

[27] Livak K.J., Schmittgen T.D. (2001). Analysis of relative gene expression data using real-time quantitative PCR and the 2^−ΔΔCT^ method. Methods.

[28] King A.M., Macrae T.H. (2015). Insect heat shock proteins during stress and diapause. Annu. Rev. Entomol..

[29] Wu P., Shang Q., Huang H., Zhang S., Zhong J., Hou Q., Guo X. (2019). Quantitative proteomics analysis provides insight into the biological role of *Hsp90* in *BmNPV* infection in *Bombyx mori*. J. Proteom..

[30] Lu Y., Li D., Ai H., Xie X., Jiang X., Afrasiyab, Zhang H., Xu J., Huang S. (2023). Glucose-regulated protein 94 facilitates the proliferation of the *Bombyx mori* nucleopolyhedrovirus via inhibiting apoptosis. Int. J. Biol. Macromol..

[31] Ruan H.Y., Zhou L., Yang L., Meng J.Y., Zhang C.Y. (2024). Functional analysis of two *SfHsp90* genes in response to high temperature and insecticide stress in *Spodoptera frugiperda* (Lepidoptera: Noctuidae). Eur. J. Entomol..

[32] Li J., Buchner J. (2013). Structure, function and regulation of the *Hsp90* machinery. Biomed J..

[33] Schopf F.H., Biebl M.M., Buchner J. (2017). The *HSP90* chaperone machinery. Nat. Rev. Mol. Cell Biol..

[34] Joshi A., Ito T., Picard D., Neckers L. (2022). The mitochondrial *HSP90* paralog *TRAP1*: Structural dynamics, interactome, role in metabolic regulation, and inhibitors. Biomolecules.

[35] Sgrò C.M., Terblanche J.S., Hoffmann A.A. (2016). What can plasticity contribute to insect responses to climate change?. Annu. Rev. Entomol..

[36] González-Tokman D., Córdoba-Aguilar A., Dáttilo W., Lira-Noriega A., Sánchez-Guillén R.A., Villalobos F. (2020). Insect responses to heat: Physiological mechanisms, evolution and ecological implications in a warming world. Biol. Rev..

[37] Zhao L., Jones W.A. (2012). Expression of heat shock protein genes in insect stress responses. Invertebrate. Surviv. J..

[38] Nguyen M.C., Tu G.H., Koprivnikar K.E., Gonzalez-Edick M., Jooss K.U., Harding T.C. (2010). Antibody responses to galectin-8, *TARP* and *TRAP1* in prostate cancer patients treated with a GM-CSF-secreting cellular immunotherapy. Cancer Immunol. Immunother..

[39] Xie S., Wang X., Gan S., Tang X., Zhu S. (2021). The mitochondrial chaperone *TRAP1* as a candidate target of oncotherapy. Front. Oncol..

[40] Hua G., Zhang Q., Fan Z. (2007). Heat shock protein 75 (*TRAP1*) antagonizes reactive oxygen species generation and protects cells from granzyme M-mediated apoptosis. J. Biol. Chem..

[41] Costa A.C., Loh S.H.Y., Martins L.M. (2013). Drosophila *Trap1* protects against mitochondrial dysfunction in a *PINK1/parkin* model of Parkinson’s disease. Cell Death Dis..

[42] Baqri R.M., Pietron A.V., Gokhale R.H., Turner B.A., Kaguni L.S., Shingleton A.W., Kunes S., Miller K.E. (2014). Mitochondrial chaperone *TRAP1* activates the mitochondrial UPR and extends healthspan in *Drosophila*. Mech. Ageing Dev..

[43] Chrétien D., Bénit P., Hyung-Ho H., Susanne K., Riyad E.K., Young-Tae C., Martin J., Jacobs H.T., Pierre R., Malgorzata R. (2018). Mitochondria are physiologically maintained at close to 50 °C. PLoS Biol..

[44] Yu H., Yang Z., Sui M., Cui C., Hu Y., Hou X., Xing Q., Huang X., Bao Z. (2021). Identification and characterization of *hsp90* gene family reveals involvement of *hsp90*, *grp94* and not *TRAP1* in heat stress response in *Chlamys farreri*. Genes.

